# The tibial insertion of the hamstring can be considered to be preserved during anterior cruciate ligament reconstruction

**DOI:** 10.3389/fsurg.2022.996289

**Published:** 2022-09-21

**Authors:** Kaibin Fang, Zhangsheng Dai, Xiaocong Lin

**Affiliations:** Department of Orthopaedic Surgery, The Second Affiliated Hospital of Fujian Medical University, Quanzhou, China

**Keywords:** hamstring, tibial insertion, anterior cruciate ligament reconstruction, arthroscope, sports medicine

## Abstract

**Background:**

Hamstring as a graft was very common in anterior cruciate ligament reconstruction surgery. Usually the hamstring muscles needed to be taken out and then woven to be used.

**Aim:**

In order to investigate whether it was beneficial for patients to preserve the transpedicular insertion of hamstring when using the hamstring as a graft for anterior cruciate ligament reconstruction.

**Methods:**

This was a retrospective study. Patients with anterior cruciate ligament injury who underwent surgery in a large hospital from January 2015 to May 2021 were included in the study. These patients underwent anterior cruciate ligament reconstruction assisted by arthroscopic. Autologous hamstring muscles were used as grafts. The tibial insertion of the hamstring were preserved during the operation were included in the observation group. The remaining patients were included in the control group. The knee joint function and operation of the two groups were compared.

**Results:**

A total of 97 patients were included in the study. There was no statistical difference between the two groups in general data including gender, age and surgical side. All the patients’ operations were successfully completed there was no significant difference in the operation time between the two groups. All patients were followed up for at least 1 year. No patients had complications such as wound infection and graft failure at the last follow-up. There was no significant difference between the two groups in Lysholm score and IKDC score before operation. Similarly, there was no significant difference between the two groups in Lysholm score and IKDC score 3 months after operation. However, the Lysholm score and IKDC score of the two groups 1 year after operation were statistically different, and the patients in the observation group had higher Lysholm score and IKDC score. After comparing the MRI images of the knee of the two groups 3 months after operation through the MRI evaluation system, compared with the patients in the control group, the patients in the observation group have higher scores, and the difference was statistically significant.

**Conclusion:**

In the knee arthroscopic assisted anterior cruciate ligament reconstruction using the hamstring as a graft, the tibial insertion of the hamstring can be preserved, which can make the patient have better function after the operation. This kind of operation leads to the increase of operation time and operation risk.

## Introduction

Anterior cruciate ligament injury was a very common disease. Generally speaking, the broken anterior cruciate ligament was difficult to heal by itself (1). Reconstructive surgery was very necessary. It is very common for an autogenous hamstring muscle to be used as a graft for arthroscopic assisted anterior cruciate ligament reconstruction (2). The fully removed and woven hamstring muscle were very suitable as grafts. However, the hamstring muscle, which is used as a graft, needs to undergo the phase of revascularization before it can fully function (3). Graft rupture is one of the most catastrophic complications at this stage (4).

In order to preserve the blood supply of the hamstring muscle as much as possible, the bone insertion point of the hamstring muscle can be considered to be preserved. In the animal model, the hamstring muscle with the dead center preserved showed excellent vitality. This was because part of its blood supply had been preserved. The probability of tendon bone healing and biomechanical strength were also improved (5).

The authors reported the results of the knee arthroscopic assisted anterior cruciate ligament surgery using the hamstring muscle as a graft, and reported the results of the surgery when the insertion point of the hamstring was preserved.

## Materials and methods

The authors conducted a retrospective study between January 2015 to May 2021. Patients with anterior cruciate ligament injury who went to a large hospital and underwent arthroscopic assisted anterior cruciate ligament reconstruction were included in the study. The exclusion criteria and inclusion criteria of the study were shown in Table 1. The choice of operation method was random. Our study was approved by the Hospital Ethics Committee.

**Table 1:** Exclusion criteria and inclusion criteria.

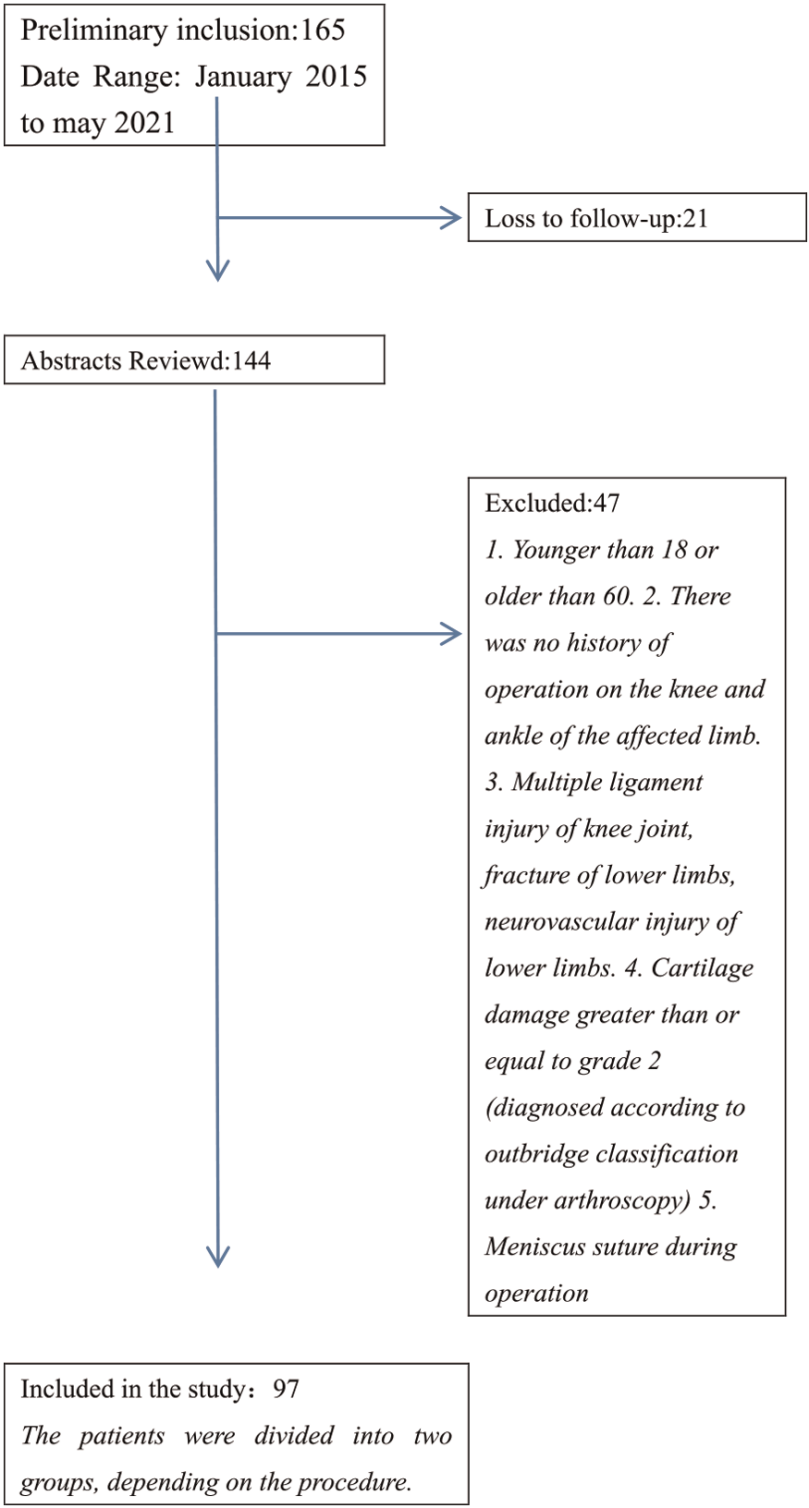

Operations were performed by the same orthopedic chief surgeon. During the operation, the patient was in supine position. An inflatable tourniquet was tied to the base of the patient's thigh. The patient underwent arthroscopic assisted anterior cruciate ligament reconstruction. The probe was the first to start. When the rupture of cruciate ligament was confirmed during operation and no other diseases such as meniscus damage and cartilage damage that need to be treated immediately are found, graft preparation can be considered. The hamstring was used as a graft.

The tendon extractor with an open end was used to cut the hamstring muscles of the patients in the observation group. The patient's knee joint was flexed to 90°. The operator cut a longitudinal incision of 2–3 cm to the distal side of the knee joint from 1.5 cm inside and half cm far from the tibial tubercle of the affected limb. The deep and superficial fascia was then dissected until the pes anserinus was exposed. The suturer's tendon membrane was incised obliquely. The gracilis muscle beneath the sartorius tendon membrane was found. Then, under the gracilis muscle, the oblique semitendinosus tendon was found. The tissue around the tendon should not be separated too much. Tibial insertion ends of semitendinosus tendon and gracilis tendon were preserved. The hip joint of the affected limb needs to be abducted when the tendon was removed. Semitendinosus and gracilis tendons were taken out through the opening. The surgeon pushed the tendon extractor toward the ischial tubercle until the tendon was disconnected.

**Figure 1:**
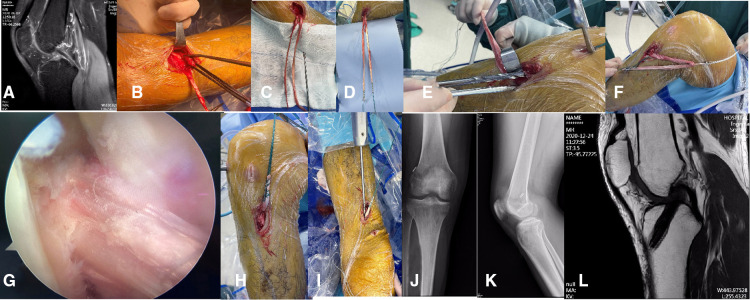
Observer patient D operation process. (**A**) Preoperative magnetic resonance imaging showed anterior cruciate ligament rupture. (**B**) The tibial insertion of the graft was preserved. (**C**) During the operation, tendon extractors with distal openings are used. (**D**) The graft is woven. (**E**) The stop of the graft is protected when the tibial tunnel is prepared. (**F**) The graft is estimated before it is placed. (**G**) With the help of arthroscopy, the grafts were observed to show good tension. (**H,I**) Interface screws were used to reinforce the graft. (**J,K**) Postoperative x-ray. (**L**) Postoperative magnetic resonance images.

After the residual muscle and soft tissue on the tendon were removed, the tendon was woven. Tendons were usually woven and folded into four strands. After the tendon was folded in half, the Endobuton plate was placed in the folded place. The graft was then placed into the knee cavity through a bone tunnel. The side with the steel plate was placed on the femoral side. When the graft was repeatedly confirmed to have reached the appropriate tightness. The interface screw was then used to fix the tendon at the tibial end. The procedure is shown in Figure 1.

The hamstring muscle in the control group were completely removed and prepared into grafts. Interface screws and plates with Endobuton plate were also used for graft fixation.

Both groups received the same rehabilitation plan. The knee protector with adjustable angle was used to limit the movement of the patient's knee. Patients can go down with the aid of crutches after operation. Within 1 week after operation, the flexion angle of the affected knee joint shall not be greater than 30°. Then adjust the brace. The knee joint of the patient can flex to 90° within 1 month after operation. Patients need to wear braces for 6 weeks. After removing the brace, the affected limb was encouraged to perform maximum knee flexion and extension training. Six months after surgery, patients were allowed to take part in some vigorous exercises, such as swimming and cycling. Nine months after the operation, the patient was allowed to participate in competitive sports activities again.

Lysholm score and IKDC score were used to evaluate the knee function of patients. Lysholm score evaluates the patient's gait, weight-bearing ability of knee joint, whether the knee joint has strangulation symptoms, stability of knee joint, pain of knee joint, swelling of knee joint, ability of patients to go upstairs and downstairs, squatting function of patients, etc. The score is between 0 and 100. The higher the score, the better the knee function (6). IKDC score is also used to evaluate knee function. Similarly, the higher the score, the better the function (7).

Patients needed to receive MRI examination 3 months after operation. The MRI image evaluation method reported by Figueroa was used to evaluate the graft condition of the two groups of patients (8). Regarding graft signal intensity, 3 parameterscould be reported: hyperintense, isointense, and hypointense. A score was assigned to each: 1, 2, and 3 points, respectively. Regarding synovial fluid presence, 2 possible findings could be reported: positive or negative. We assigned 1 point for positive and 2 points for negative synovial fluid at the graft-tunnel interface (8).

### Statistical analysis

SPSS statistical package program (SPSS 19.0 version;SPSS Inc., Chicago, Illinois, USA) was used for statisticalanalysis. Data were presented as mean ± SD (range), median (range), or *n* (%). *χ*^2^-test (categorical data) or Student's *t*-test (continuous data) was used to compare theresults from two groups. And a *P*-value of <0.05 was considered significantly different.

## Results

A total of 97 patients were included in the study. There was no statistical difference between the two groups in general data including gender, age and surgical side. All the patients’ operations were successfully completed There was no significant difference in the operation time between the two groups.

All patients were followed up for at least 1 year. No patients had complications such as wound infection and graft failure at the last follow-up.

There was no significant difference between the two groups in Lysholm score and IKDC score before operation. Similarly, there was no significant difference between the two groups in Lysholm score and IKDC score 3 months after operation. However, the Lysholm score and IKDC score of the two groups 1 year after operation were statistically different, and the patients in the observation group had higher Lysholm score and IKDC score.

After comparing the MRI images of the knee joints of the two groups 3 months after operation through the MRI evaluation system, compared with the patients in the control group, the patients in the observation group have higher scores, and the difference is statistically significant. Specific statistical data were shown in Table 2.

**Table 2:** Demographic data and clinical outcomes.

Variable	Observation group (*n* = 35)	Control group (*n *= 62)	Statistic (*T*-value/*χ*^2^-value)	*P*-value
Gender (male/female)	25/10	50/12	1.08	0.30
Age (year)	33.17 ± 7.25	30.77 ± 6.15	1.73	0.09
The operation time (minute)	75.36 ± 9.13	73.96 ± 13.35	0.55	0.58
Surgical side (left/right)	16/19	30/32	0.06	0.80
Lysholm score before operation	51.17 ± 7.95	50.77 ± 9.12	0.22	0.82
Lysholm score 3 months after surgery	70.55 ± 6.13	71.03 ± 7.33	0.33	0.74
Lysholm score one tear after surgery	85.11 ± 5.77	81.29 ± 6.08	3.03	<0.01
IKDC score before operation	50.72 ± 6.33	51.26 ± 7.75	0.35	0.72
IKDC score 3 months after surgery	70.15 ± 5.66	69.73 ± 7.25	0.30	0.77
IKDC score one tear after surgery	83.95 ± 9.36	80.25 ± 7.05	2.20	0.03
Magnetic resonance imaging evaluation	3.15 ± 0.55	2.95 ± 0.35	2.18	0.03

Values are presented as mean ± SD.

## Discussion

In the authors’ report, the hamstring muscle as a graft was used for anterior cruciate ligament reconstruction as an effective graft while retaining the insertion point. Of course, in order for the insertion point to be preserved, the preparation of the graft must be carried out on the patient's knee joint. Including tendon processing and weaving. Therefore, the operation time of patients in the observation group was longer, but this was not statistically significant. This may be related to the use of tendon extractors with rings. When the tendon is treated by the assistant, the bone tunnel can be prepared by the surgeon at the same time.

Compared with patients in the control group, patients in the observation group had certain advantages in knee function score, especially 1 year after operation. When the patients in the observation group received imaging reexamination after operation, the grafts on the magnetic resonance imaging also showed better vitality. These results confirm that the hamstring muscle as a graft can play a better role, and stop point is preserved at its tibial insertion. Whether the tibial side of the graft is fully fixed will play a decisive role in the failure of anterior cruciate ligament surgery (9). Compared with bone to bone healing, the speed and ability of tendon to bone healing are relatively poor (10). Before the tendon-bone healing, the graft may come out of the bone tunnel, leading to the failure of the operation (11). The preservation of the tibial insertion of the graft can make the graft have better failure load and pullout resistance (12). Some scholars have reported that when no other internal plant for fixation is used on the tibial side, the hamstring muscle as a graft can still play its full role when the insertion point is retained, and there is no case that the graft leaves the bone tunnel (13).

However, in the author's study, the interface screw is still used to fix the tibial side of the graft. This is for many reasons. First, the prepared graft is woven from two tendons folded in half. This resulted in the free ends of two tendons in the prepared graft on the tibial side. If the graft is not fixed, the wiper effect and bungee effect may occur (14). Second, the retention of the dead center will keep the hamstring muscles under high-intensity tension. When the knee joint is active, goose foot bursitis may occur. Third, it is also to ensure the stability of the graft to the greatest extent and prevent graft failure secondary to the hissing of the tibial stop of the hamstring muscle.

In the author's study, the interface screw was selected as the fixation of the tibial side, rather than the screw or FOOTPRINT Ultra, or Endobuton plate. Interface screws used for tibial fixation of grafts can significantly increase the initial pullout resistance of grafts (15). Compared with traditional screws, the use of interface screws does not affect the blood flow of the hamstring muscle as a graft. There was no change in signal intensity of the hamstring used as grafts on MRI images (16). This indirectly shows that the ratio between blood composition and water content of hamstring muscles has not changed due to the use of interface screws.

The graft needs to undergo revascularization, tendon bone healing and tendon ligamentization before it can play its full role (17). The method reported by the authors seems to allow the hamstring muscle as a graft to skip the revascularization stage. At the same time, if the blood supply of hamstring is still preserved, better tendon bone healing may be achieved. The better knee function score and MRI evaluation score of patients in the observation group may confirm this conjecture.

More cases and longer follow-up are still needed to confirm our results. Through the existing research, the author believes that in the knee arthroscopic assisted anterior cruciate ligament reconstruction using the hamstring as a graft, the tibial insertion of the hamstring can be preserved, which can make the patient have better function after the operation. This kind of operation leads to the increase of operation time and operation risk.

## Data Availability

The original contributions presented in the study are included in the article, further inquiries can be directed to the corresponding author.
